# Ahorros para el sistema de salud colombiano con la financiación externa de estudios clínicos oncológicos

**DOI:** 10.7705/biomedica.7239

**Published:** 2025-03-28

**Authors:** Sandra Aruachan, Manuel González, Danis M. Rojas, Javier Ospina, Santiago Duque-Varela, Andrés Ángel Castaño

**Affiliations:** 1 Unidad de Estudios Clínicos, Clínica IMAT Oncomédica Auna, Montería, Colombia Clínica IMAT Oncomédica Auna Clínica IMAT Oncomédica Auna Montería Colombia; 2 Servicio de Oncología Clínica, Clínica IMAT Oncomédica Auna, Montería, Colombia Clínica IMAT Oncomédica Auna Clínica IMAT Oncomédica Auna Montería Colombia; 3 Área de Estrategia de Modelos de Aseguramiento, Access Co S.A.S., Pereira, Colombia Access Co S.A.S. Access Co S.A.S. Pereira Colombia

**Keywords:** sistemas de salud, ahorro de costo, estudio clínico, neoplasias, industria farmacéutica, Health systems, cost savings, clinical trial, neoplasms, drug industry

## Abstract

**Introducción.:**

El gasto en medicamentos para tratar el cáncer aumentará entre el 9 y el 12 % anual hasta el 2025. Para los sistemas de salud de los países con un ingreso medio-alto, como Colombia, y con una tendencia creciente de nuevos casos de cáncer, la investigación clínica puede contribuir al uso eficiente de los recursos que tiene el sistema sin menoscabar la oportunidad y la calidad de la atención.

**Objetivo.:**

Calcular el ahorro generado al sistema de salud colombiano por la implementación de estudios clínicos para cáncer con financiación externa.

**Materiales y métodos.:**

Se desarrolló un estudio observacional, longitudinal, descriptivo y retrospectivo, basado en el análisis de las historias clínicas de participantes de estudios clínicos realizados entre el 2016 y el 2022 en la Clínica IMAT Oncomédica Auna, Colombia.

**Resultados.:**

El ahorro en medicamentos oncológicos para el sistema de salud por financiación externa fue de USD $1’526.320 y el ahorro promedio ponderado mensual por paciente fue de USD $3.257. La participación de los pacientes con cáncer de mama en los estudios clínicos controlados aleatorizados (n = 138) representó el 24 % (USD $369.363) del total de los ahorros. La financiación externa de medicamentos oncológicos para los participantes con cáncer en estadio clínico IV y III, representó el 41,7 % (USD $636.475) y el 31,06 % (USD $473.159), respectivamente, del ahorro total para el Sistema General de Seguridad Social en Salud de Colombia.

**Conclusión.:**

La participación de los pacientes con cáncer en los estudios clínicos evitó costos al sistema de salud de Colombia. El ahorro fue mayor en las mujeres con cáncer de mama y en aquellos pacientes con cáncer en estadio clínico IV.

## Abstract (en)

**Introduction.:**

Spending on drugs to treat cancer will increase by 9-12% annually until 2025. For health systems in high and middle-income countries -such as Colombia- and with an increasing trend of new cancer cases, clinical research can contribute to the efficient use of the system resources available without undermining the timeliness and quality of healthcare.

**Objective.:**

To calculate the savings generated to the Colombian health system by the implementation of externally funded clinical trials for cancer.

**Materials and methods.:**

We conducted an observational, longitudinal, descriptive, and retrospective study analyzing participant’s medical records of clinical trials between 2016 and 2022 at the *Clínica IMAT Oncomédica Auna*, Colombia.

**Results.:**

The total savings to the health system for external financing of oncology drugs was USD $1,526,320, and the monthly weighted average savings per patient was USD $3,257. The participation of breast cancer patients in randomized controlled clinical trials (n = 138) accounted for 24% (USD $369,363) of the total savings. Participants with clinical stage IV and III accounted for 41.7% (USD $636,475) and 31.06% (USD $473,159), respectively, of the total savings to the general social security health system in Colombia from external financing of oncological drugs.

**Conclusion.:**

The participation of cancer patients in clinical trials mitigated costs to the Colombian health system, especially in women with breast cancer and in those patients with clinical stage IV of the disease.

En el 2019, el gasto global en salud fue de USD $8,5 trillones, más del doble, del gasto reportado en el 2000 (USD $4.2). Según la Organización Mundial de la Salud (OMS), aproximadamente el 60 % del gasto en salud proviene de fuentes gubernamentales y, el 40 %, de fuentes locales de financiación privada ^1^.

En Colombia, el gasto en salud viene en aumento y presenta cambios en su composición. Para el 2013, el gasto general en salud del gobierno fue de USD $11.381 millones y, cinco años después, llegó a USD $17.487 millones. Este gasto comprende la atención en salud prestada por las entidades del gobierno en los distintos niveles y, por los agentes de seguridad social en salud u otros, con sistemas de financiamiento contributivo y obligatorio ^2^.

De hecho, los esquemas de seguridad social en salud financiados por el gobierno general para el 2013 representaron el 63 % del gasto en salud, mientras que para el 2018 representaron el 68,3 % ^2^. Dichos esquemas están conformados por los regímenes contributivo y subsidiado del Sistema General de Seguridad Social en Salud, y los regímenes especiales y de excepción integrados por las fuerzas armadas, los maestros y los trabajadores y pensionados de la Empresa Colombiana de Petróleos (Ecopetrol) ^2^.

También, durante este período (2013-2018), las atenciones en salud representaron el 89,2 % del total del gasto corriente en salud y, los gastos administrativos, el 10,8 %. El gasto corriente en salud corresponde al consumo final total de bienes y servicios de salud por parte de las unidades institucionales residentes en el país ^2^.

Aunado a lo anterior, la Organización Mundial de la Salud (OMS), en su último informe sobre gasto global en salud, señaló que para los países con ingresos medios y bajos, como Colombia, las enfermedades no transmisibles -incluyendo las oncológicas- representan el 26 % del gasto total en salud ^1^. En ese sentido, el informe del *IQVIA Institute for Human Data Science* sobre la tendencia del uso de medicamentos y el gasto global, señala que el gasto de medicamentos utilizados en el tratamiento del cáncer aumentará entre el 9 y el 12 % anualmente hasta el 2025, lo cual supone un gasto adicional de USD $106 billones para un total de USD $260 billones en el 2025 ^3^.

Según el Análisis de la Situación de Salud de Colombia (ASIS) más reciente, en el 2021 las neoplasias fueron la tercera causa de mortalidad en la población general y causaron el 13,56 % de las muertes ^4^. Asimismo, el último estudio de la carga mundial de la enfermedad evidenció que las neoplasias malignas, como el cáncer colorrectal, de pulmón, estómago, mama, próstata e hígado, se mantienen en el grupo de las principales causas de años de vida ajustados por discapacidad (*Disability-Adjusted Life Years*, DALYs) para personas mayores de 50 años en las últimas tres décadas ^5^.

Por otra parte, la investigación clínica en seres humanos en Colombia ha crecido en los últimos 10 años, lo cual ha permitido el aumento y el reconocimiento a nivel mundial de los centros de investigación que desarrollan estudios clínicos en el país por sus estándares de calidad ^6^. Según la Asociación Colombiana de Centros de Investigación Clínica (ACIC), para el 2022, en Colombia se reportaron 135 centros de investigación clínica certificados por el Instituto Nacional de Vigilancia de Medicamentos y Alimentos (INVIMA) ^7^.

Los estudios clínicos que más se desarrollan en Colombia son los de fase II, III y IV. Estos, generalmente, son patrocinados por la industria farmacéutica o por las organizaciones de investigación por contrato. Según clinicaltrials.gov -la base de datos de estudios clínicos administrada por los *National Institutes of Health* (NIH) de los Estados Unidos-, Colombia tiene registrados 1.769 estudios, equivalentes al 0,39 % del total de estudios clínicos realizados a nivel mundial ^8^.

Según la ACIC, la ejecución de estudios clínicos podría estar generando al país 3.400 empleos directos y aportando al producto interno bruto cerca de COP $250.000 millones ^7^. Sin embargo, se desconoce el dato exacto de la inversión en investigación clínica para las enfermedades oncológicas y del ahorro generado al Sistema General de Seguridad Social en Salud por la participación de los pacientes con cáncer en los estudios clínicos financiados por la industria farmacéutica y los organismos o agencias internacionales.

Es importante destacar que los motivos fundamentales que justifican la realización de investigaciones relacionadas con la salud en seres humanos, son el valor social y científico de la investigación. El valor social se refiere al impacto de la información producida por un estudio y, el valor científico, a la capacidad de un estudio de generar información confiable y válida, útil en la toma de decisiones que tendrán consecuencias importantes sobre la salud de las personas y la salud pública ^9^.

En este sentido, uno de los beneficios esperados de la investigación en salud es la construcción de capacidades, entendida como el compromiso multilateral e interinstitucional de generar, a partir de la cooperación internacional, la infraestructura y el entrenamiento de profesionales y científicos locales; otro beneficio es el fortalecimiento de las condiciones estructurales que permitan el fomento de la autonomía local y el desarrollo sostenible de los países no industrializados. Más aún, la construcción de capacidades en investigación es uno de los medios más eficaces, sostenibles y costo-efectivos para la promoción de la salud y el desarrollo colaborativo ^10^.

La Clínica IMAT Oncomédica Auna es una organización del sector salud dedicada a la prestación de servicios especializados en hematología y oncología. Fue fundada en Montería en el 2003 y presta sus servicios a los pacientes con diagnóstico de cáncer provenientes de distintas aseguradoras de Córdoba y diferentes departamentos de la Costa Atlántica y del país. Tiene como premisa brindar un trato humanizado, individualizado, oportuno, integral y continuo a sus pacientes.

La clínica tiene un centro de investigación propio, certificado por el INVIMA en buenas prácticas clínicas. En los últimos cinco años, este centro atendió, aproximadamente, a 20.000 pacientes con diagnóstico de algún tipo de cáncer.

El objetivo de este estudio fue estimar el ahorro generado a las finanzas del sistema general de salud colombiano, con base en la implementación de estudios clínicos para enfermedades oncológicas con financiación externa.

## Materiales y métodos

### Diseño del estudio

Se realizó un estudio observacional, longitudinal, descriptivo y retrospectivo, basado en el análisis de las historias clínicas de los participantes en estudios clínicos controlados aleatorizados llevados a cabo entre enero del 2016 y junio del 2022, en la Clínica IMAT Oncomédica Auna de Montería, Colombia.

### Selección de estudios y pacientes

En el estudio se incluyeron los pacientes oncológicos con edad mayor o igual a 18 años, que participaron en estudios clínicos controlados aleatorizados para el tratamiento del cáncer (de cualquier tipo), financiados en su totalidad por la industria farmacéutica u organizaciones públicas o privadas promotoras de investigaciones en oncología clínica. Según el protocolo de investigación de cada patrocinador, los pacientes no podían participar simultáneamente en otro estudio. Los estudios clínicos controlados aleatorizados evaluados fueron aquellos con diseño de doble ciego, pero ya desenmascarado por finalización del estudio.

Se excluyeron del análisis de costos aquellos estudios sin reclutamiento de pacientes, los clínicos controlados aleatorizados de fase 4 (posteriores a la comercialización) en los que el tratamiento no era financiado externamente y los pacientes que presentaron fallas de tamizaje.

El Comité de Ética de la Clínica IMAT Oncomédica Auna aprobó el protocolo del estudio.

### Extracción y análisis de datos

De cada historia clínica, se obtuvieron los datos del principio activo en investigación y el comparador, las dosis y la duración del tratamiento. También, se analizó el uso de los servicios de salud (consultas médicas especializadas, imágenes diagnósticas y pruebas de laboratorio) de acuerdo con el protocolo de investigación aprobado por el comité de ética. Otras variables analizadas fueron edad, sexo, ciudad de residencia y régimen de afiliación al Sistema General de Seguridad Social en Salud de Colombia.

Se usó una metodología de análisis econométrico de recopilación en archivos planos. Cada variable fue evaluada en términos de completitud, conformidad, consistencia, precisión, duplicación e integridad. Estas seis dimensiones permitieron identificar y separar los defectos de los datos y así, evaluar el alcance de la base de datos. En esta fase, también se garantizó que las codificaciones y atributos estuvieran plenamente homologados con los datos de referencia (Divipola, CIE-10, etc.). El análisis de los datos se hizo mediante el software R, versión 4.3.0, y se editaron con R Studio, versión 12.0 +353, y Tableau, versión 2022.1.1.

### Cálculo del ahorro

El ahorro se definió como el gasto que tendría que haber pagado la empresa administradora del plan de beneficios de salud en caso de que la dispensación de los medicamentos y la prestación de otros servicios médicos no hubieran sido financiadas externamente. En el caso de los estudios clínicos controlados aleatorizados en los que se utilizaron medicamentos experimentales cuyo costo no estaba disponible, el costo evitable por dispensación de medicamentos se calculó con base en el fármaco comparador.

Las tarifas se estimaron mediante «microcosteo» o *bottom-up*. Esta metodología utiliza las tarifas promedio del mercado para los servicios contratados y los recursos utilizados en el tratamiento de pacientes con la condición de salud de interés. Luego, estos costos son agregados para obtener una estimación del impacto económico del tratamiento ^11^^,^^12^.

Se tuvieron en cuenta todos los servicios solicitados por los pacientes en el tratamiento integral, entre ellos: consulta especializada, medicamentos, laboratorios y hospitalizaciones en salas generales y en unidades de cuidados intensivos. Las tarifas del mercado son las establecidas entre la aseguradora y la entidad prestadora de servicios de salud en el momento de la prestación del servicio.

En el estudio original, se expresaron los ahorros en pesos colombianos (COP), pero para esta versión, se hizo la conversión a dólares estadounidenses (USD) con una tasa promedio representativa del mercado de COP $3.327,90 por cada dólar. El promedio del peso colombiano por dólar en el periodo de los 79 meses de análisis fue de COP $3.327,90 (Banco de la República de Colombia).

Respecto a la tasa de inflación, todos los cálculos se hicieron en precios corrientes, tomando como referencia una tasa promedio de inflación anual del 4,4 % (Banco de la República de Colombia).

Los resultados se expresaron como frecuencias absolutas o relativas (porcentaje). También, se calcularon medidas de tendencia central (media y mediana) y de dispersión [desviación estándar y rango intercuartílico (RIC) según la distribución de los datos.

## Resultados

Durante el período considerado, el centro de investigación llevó a cabo 52 estudios clínicos controlados aleatorizados, de los cuales 35 (67 %) incluyeron pacientes oncológicos y con financiación de los laboratorios farmacéuticos. Se excluyeron 1.060 pacientes por fallas en la selección según el protocolo del estudio clínico. Al final, en el análisis de costos ahorrados se incluyeron 411 pacientes. La mayoría de ellos estaban entre los 40 y los 64 años (n = 207; 50,4 %). El 67,6 % (n = 278) de los pacientes del estudio eran mujeres (n = 278) y el 32,4 % (n = 133) eran hombres (cuadro 1).

**Cuadro 1:** Características de los pacientes incluidos en el estudio (N = 411)

Variable		n (%)
Sexo		
	Mujer	278 (67,6)
	Hombre	133 (32,4)
Grupos de edad (años)		
	20-39	44 (10,7)
	40-64	207 (50,4)
	Más de 65	160 (38,9)
Estadio clínico en el momento del ingreso al estudio clínico		
	I	47 (11,4)
	II	64 (15,6)
	III	99 (24,1)
	IV	201 (48,9)
Tipo de cáncer		
	Mama	138 (33,6)
	Próstata	36 (8,7)
	Estómago	34 (8,3)
	Ginecológico^†^	25 (6,1)
	Pulmón	16 (3,9)
	Otros^††^	162 (39,4)

^†^ Incluye pacientes con cáncer de cuello uterino y ovario.

^††^ Incluye pacientes con cánceres de baja incidencia en la población local, como cáncer de vejiga, glioblastoma, de esófago, piel o endometrio, linfoma de Hodgkin y cáncer de origen primario desconocido.

Veintiocho de los estudios incluidos eran de fase III, 5 de fase II, y 1 de fase I, y un estudio combinó las fases II y III. El 48,9 % (n = 201) de los pacientes incluidos en los estudios clínicos controlados aleatorizados se clasificaron con cáncer en estadio clínico IV al momento de su ingreso, el 24,1 % (n = 99) en estadio clínico III, el 15,6 % (n = 64) en estadio clínico II y 11,4 % (n = 47) en estadio clínico I (cuadro 1).

En cuanto al tipo de cáncer, de 27 estudios llevados a cabo: 10 fueron de mama (138 pacientes), 3 de estómago (34 pacientes), 5 de próstata (36 pacientes), 5 de pulmón (16 pacientes) y 4 de tumores ginecológicos (25 pacientes) (cuadro 1).

### Ahorros

El costo ahorrado al sistema de salud colombiano por financiación externa de medicamentos oncológicos durante el período del estudio, fue de USD $1’526.320. La mediana estimada de costos ahorrados por año fue de USD $2.833 y el costo evitado promedio ponderado mensual por paciente fue de USD $3.257.

La participación de los pacientes con cáncer de mama en los estudios clínicos controlados aleatorizados (n = 138) representó el 24 % (USD $369.363) del ahorro. Por otra parte, los pacientes con cáncer de pulmón (n = 16) representaron el 7,2 % (USD $109.488) del total ahorrado al Sistema General de Salud de Colombia (cuadro 2). El ahorro promedio ponderado mensual de los pacientes con cáncer de mama fue de USD $4.155.

**Cuadro 2:** Costos ahorrados por estadio clínico de la enfermedad en el momento del diagnóstico y tipo de cáncer diagnosticado

Estadio clínico		Ahorro total (USD)	Ahorro total (%)
I		$ 129.737	8,5
	Cáncer de mama	$ 64.189	49,5
	Cáncer de ovario	$ 10.796	8,3
	Cáncer de pulmón	$ 4.812	3,7
	Otros tipos de cáncer	$ 49.940	38,5
II		$ 285.422	18,7
	Cáncer de mama	$ 113.880	39,9
	Cáncer de estomago	$ 6.837	2,4
	Otros tipos de cáncer	$ 164.705	57,7
III		$ 473.159	31,06
	Cáncer de mama	$ 115.847	24,5
	Cáncer de ovario	$ 14.306	3,0
	Cáncer de estómago	$ 13.883	2,9
	Otros tipos de cáncer	$ 329.123	69,6
IV		$ 636.475	41,7
	Cáncer de pulmón	$ 104.676	16,4
	Cáncer de mama	$ 75.447	11,9
	Cáncer de ovario	$ 63.976	10,1
	Cáncer de estómago	$ 58.203	9,1
	Otros tipos de cáncer	$ 334.173	68,9
Total		$ 1.526.320	100

Respecto a los costos ahorrados según el estadio clínico en el momento del diagnóstico del cáncer, los participantes en estudios clínicos controlados aleatorizados clasificados en el estadio clínico IV de la enfermedad representaron el 41,7 % (USD $636.475) del total ahorrado al Sistema General de Seguridad Social en Salud por financiación externa de medicamentos oncológicos, mientras que aquellos clasificados en estadio clínico III, representaron el 31,06 % (USD $473.159). Ciertamente, los pacientes con cáncer de pulmón, mama, ovario o estómago, fueron los que generaron mayores ahorros en dichos estadios clínicos. Con los pacientes con cáncer de estómago, el ahorro promedio ponderado mensual fue de USD $2.745 y, con aquellos con cáncer de ovario, fue de USD $5.075. Los costos evitados según el estadio clínico del cáncer en el momento del diagnóstico, se muestran en el cuadro 2.

### Ahorros según servicios de salud utilizados

Respecto a los servicios de salud requeridos por los pacientes, que fueron financiados por el patrocinador del estudio clínico, la administración de quimioterapia y la hospitalización fueron los más utilizados y con mayor participación en el costo del tratamiento, con una mediana de USD $1.933 (481 - 5.144) y USD 654 (79 - 2.797), respectivamente. Al final, estos ahorros para el sistema de salud se debieron a la financiación externa (cuadro 3).

**Cuadro 3:** Costos ahorrados según los servicios de salud utilizados

Servicio	Ahorros (USD) Mediana	RIC (USD)
Administración de quimioterapia	$ 1.933	$ 481 a $ 5.144
Imagenología	$ 451	$ 199 a $ 994
Hospitalización	$ 654	$ 79 a $ 2.797
Cirugía	$ 469	$ 28 a $ 1.825

RIC: rango intercuartílico; USD: dólares estadounidenses

### Ahorros según sexo y grupos de edad

La atención de mujeres generó un mayor ahorro promedio por paciente (USD $4.397), en comparación con la de los hombres (USD $2.286). En efecto, el costo total evitado por la participación de mujeres en los estudios clínicos controlados aleatorizados (n = 278) fue de USD $1’222.325 y para los hombres (n = 133) fue de USD $303.995.

En cuanto a los grupos etarios, en la atención de pacientes entre los 55 y los 59 años se reportó el mayor ahorro para el sistema de salud (USD $250.193), lo cual representó el 16,4 % del costo total ahorrado durante el período de análisis.

**Figura 1:**
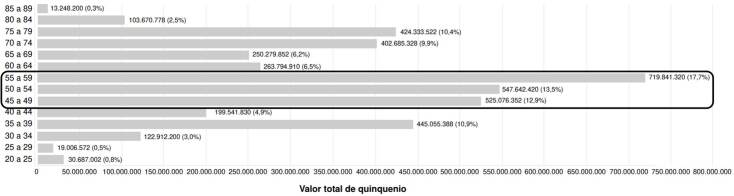
Costos ahorrados (en pesos colombianos, COP) en la atención de las mujeres participantes en estudios clínicos controlados aleatorizados, financiados externamente. Se muestran por grupo de edad.

Se hace énfasis en el análisis de los ahorros en las mujeres que participaron en los estudios clínicos aleatorizados. En los grupos de aquellas entre los 55 y los 59 años (17,7 %; USD $216.305), los 50 y los 54 años (13,5 %; USD $164.561) y los 45 y los 49 años (12,9 %; USD $157.780), se encontraron los mayores ahorros. Estos valores significaron el 44 % (USD $238.156) del total del ahorro generado al sistema de salud por su participación en los ensayos clínicos controlados aleatorizados (figura 1).

## Discusión

En el presente estudio, se encontró que la atención de 411 pacientes con cáncer -incluidos en los estudios clínicos patrocinados por laboratorios farmacéuticos- generó un ahorro al Sistema General de Seguridad Social en Salud de Colombia de USD $1’526.320 en cinco años. Esto significó un ahorro promedio ponderado mensual por paciente, de USD $3.257. El ahorro fue mayor en casos de cáncer de mama, particularmente en aquellos con el estadio clínico IV de la enfermedad en el momento del diagnóstico y en mujeres entre los 45 y los 59 años.

De acuerdo con la revisión bibliográfica realizada en varias bases de datos (PubMed, LILACS y Google Scholar), es posible afirmar que este estudio es el primero en el país que reporta los costos ahorrados al sistema nacional de salud por la participación de pacientes con cáncer en estudios clínicos financiados por la industria farmacéutica. Estos se llevaron a cabo en una institución oncológica de cuarto nivel ubicada en una ciudad intermedia, resaltándose su ubicación en la Región Caribe de Colombia.

El cáncer de mama es el de mayor incidencia en el mundo, sobre todo, en mujeres. De acuerdo con el Observatorio Global del Cáncer, en el 2022, el 24 % de los nuevos casos de cáncer en ellas fue el de mama y, para el 2025, se estima un aumento del 113 % en las mujeres adultas colombianas ^13^^,^^14^. En su informe del 2022, la Cuenta de Alto Costo -el organismo nacional que coordina y revisa la gestión del riesgo de las enfermedades de alto costo por los diferentes actores del Sistema General de Seguridad Social en Salud- señaló que el cáncer de mama sigue siendo el más común entre las mujeres colombianas, con el 29 % del total de nuevos casos. Estos casos incidentes ocurrieron en mujeres con una mediana de edad de 59 años (RIC = 49 - 68) y la mayoría se clasificaron en estadios clínicos II y III en el momento del diagnóstico ^15^.

El aumento de las opciones terapéuticas para el tratamiento del cáncer de mama en sus diferentes estadios clínicos, ha mejorado la supervivencia en estas pacientes ^16^^-^^19^. Sin embargo, a la par de estos beneficios, se han incrementado los costos de atención. En la revisión sistemática de Sun et al., se reportó que, en promedio, los costos de su tratamiento en estadios II (32 %), III (95 %) y IV (109 %) fueron mayores que los de la enfermedad en estadio I ^20^. Estos resultados son coherentes con los hallazgos del presente estudio, pues el grupo en el que se identificó mayor ahorro para el sistema de salud por su participación en estudios clínicos controlados aleatorizados fue el de pacientes clasificados con estadio clínico IV en el momento del diagnóstico de cáncer.

Se han publicado varios estudios que analizan los costos evitados al sistema de salud y a los centros de atención médica, por la participación de los pacientes en estudios clínicos controlados aleatorizados financiados por la industria farmacéutica ^11^^,^^21^^-^^25^. Los ahorros, reportados por estos estudios internacionales, oscilan entre USD $104,90 y USD $419.000 por año; y, para los centros de atención médica, el ahorro promedio anual puede ser de hasta USD $58.000 ^26^.

En tal sentido, los resultados del presente estudio se encuentran dentro del rango de costos evitados al sistema de salud por la participación de pacientes en estudios clínicos patrocinados por la industria farmacéutica. No obstante, la comparación de estos resultados con los de otras investigaciones similares puede ser complicada, debido a la variabilidad de los precios de los medicamentos entre países.

Otro hallazgo interesante del presente estudio fue que la administración de la quimioterapia fue el aspecto que más contribuyó al ahorro de gastos para el sistema de salud. Esto es coherente con el estudio de costos directos de la atención de cáncer de mama en Colombia, realizado por Gamboa *et al*., quienes observaron que, excepto por el cáncer *in situ*, la quimioterapia es el componente principal de los costos de atención del paciente con cáncer de mama en los demás estadios de la enfermedad, lo que explica desde el 75,0 hasta el 87,6 %, según el estadio ^27^.

Para los sistemas de salud de los países con ingresos medios a altos, como Colombia, y con una tendencia al aumento de nuevos casos de cáncer ^15^^,^^28^, la investigación clínica desarrollada bajo altos estándares de calidad, enfocada en problemas de salud pública o enfermedades con alta carga y centrada en el paciente, se convierte en un potenciador de los sistemas de salud. Más aún, la investigación clínica puede contribuir al uso eficiente de los recursos que tiene el sistema de salud, sin menoscabar la oportunidad y la calidad de la atención.

De hecho, la publicación reciente de Cardona-Zorrilla *et al*. ^29^, sobre la participación en estudios clínicos oncológicos patrocinados y su impacto presupuestal en el sistema de salud colombiano, confirma el efecto de la investigación clínica en la eficiencia de los recursos económicos del sistema de salud. Según los autores, si, al menos, el 25 % de los pacientes con tumores en estadio avanzado, de alta prevalencia y carga económica, como aquellos con cáncer de próstata, de estómago, de mama (HER2 positivo o triple negativo) y mieloma múltiple, hubieran sido incluidos en estudios clínicos controlados aleatorizados de fase III durante el 2023, el Sistema de Salud Colombiano hubiera ahorrado hasta el 4 % del total del gasto en medicamentos (USD $5 billones).

Cabe resaltar que los pacientes participantes en estudios clínicos reciben directamente los beneficios no económicos de los estudios, como el acceso rápido a medicamentos innovadores, el mejoramiento de la calidad asistencial derivado de la transferencia tecnológica del desarrollo de los estudios clínicos y el crecimiento profesional del personal implicado en las investigaciones clínicas. En otras palabras, la investigación clínica integrada a los sistemas de salud trae utilidad social para los países que acogen e impulsan este tipo de alianzas ^21^^,^^30^^,^^31^.

Sumado a lo anterior, el *National Cancer Institute* (NCI) de los Estados Unidos ha propuesto que las investigaciones en economía de la salud del cáncer sean transdisciplinarias mediante la colaboración de investigadores de diferentes disciplinas (epidemiólogos, investigadores de servicios de salud, científicos de datos, formuladores de políticas y defensores de pacientes) y que incluyan la interacción entre las redes de investigación existentes, como las redes cooperativas de estudios clínicos. Esto permitiría que las investigaciones incorporen datos de diferentes fuentes, lo cual es fundamental para la generalización de los hallazgos. Además, el NCI ha enfatizado que las investigaciones en economía de la salud oncológica deben contar siempre con un enfoque del mundo real.

En consecuencia, este tipo de investigación será útil para cubrir brechas en áreas poco estudiadas, como el efecto del diseño de los seguros de salud en la atención y del cáncer; además, no solo ayudará a reducir los costos de bolsillo y las dificultades financieras para las personas con cáncer, sino también, a disminuir el uso de servicios de atención de bajo valor, innecesarios y potencialmente dañinos ^32^.

En ese sentido, el presente estudio está alineado con la propuesta del NCI sobre los próximos desafíos de la investigación en economía de la salud del cáncer y su impacto en la atención oncológica y el desarrollo e implementación de políticas públicas relacionadas.

Una limitación de este estudio es que el ahorro por dispensación de medicamentos se calculó en función de los fármacos comparadores, lo cual subestima el impacto económico de los estudios clínicos sobre el sistema de salud. Por lo tanto, para mayor precisión de la estimación del impacto económico, es pertinente actualizar el cálculo del costo evitado después de que los patrocinadores levanten el cegamiento del estudio y se establezca el costo de comercialización de los medicamentos innovadores dentro del sistema de salud.

Otra limitación del estudio fue no incluir la etapa de extensión de los estudios clínicos controlados aleatorizados en el cálculo de los costos evitados. La etapa de extensión es subsecuente a la finalización del estudio clínico y, durante este período, el patrocinador del estudio continúa suministrando el tratamiento innovador a los participantes hasta que sea aprobado por la agencia nacional reguladora de medicamentos y comience su comercialización en el sistema de salud. Ciertamente, esta etapa es importante para estimar el ahorro que supone al sistema de salud la participación en estudios clínicos financiados externamente.

Además, las pruebas de diagnóstico (biológicas, moleculares o imagenológicas) utilizadas por los pacientes que no fueron incluidos en los estudios clínicos, no se contemplaron en el análisis de los costos ahorrados. Esto subestima el impacto económico de la participación en los estudios clínicos controlados aleatorizados.

Una fortaleza del presente estudio fue incluir los servicios médicos requeridos por el paciente durante su participación en el estudio clínico -y que fueron cubiertos por el patrocinador- en el cálculo de los costos ahorrados al Sistema General de Seguridad Social en Salud. Estos abarcan hospitalizaciones, administración de quimioterapia, imágenes diagnósticas y cirugía, y son un componente importante del costo de la atención médica.

En resumen, la atención brindada por la Clínica IMAT Oncomédica Auna a 411 pacientes con cáncer, que participaron en los estudios clínicos patrocinados por la industria farmacéutica, le ahorró al sistema de salud colombiano USD $1’526.320 en cinco años. Estos resultados son, en sí mismos, evidencia para continuar promoviendo la investigación clínica en Colombia, ya que muestran empíricamente el beneficio público y social de la alianza entre los sistemas de salud y la industria farmacéutica.

Los investigadores de este estudio consideran beneficioso para la sostenibilidad del sistema de salud colombiano, el realizar investigaciones que den continuidad al presente estudio y que incluyan otros tipos de cáncer o enfermedades relevantes para el sistema de salud por su prevalencia y carga económica (por ejemplo, la enfermedad pulmonar obstructiva crónica, la diabetes o las enfermedades neurodegenerativas). Sobre todo, que los nuevos estudios se desarrollen mediante colaboraciones entre los centros de investigación clínica del país y los diferentes actores del sistema de salud, como las asociaciones de pacientes, los prestadores de servicios médicos hospitalarios y ambulatorios, y las aseguradoras de servicios de salud.

Además, es conveniente que las futuras investigaciones analicen otro tipos de costos, como los indirectos que asumen los participantes en los estudios clínicos y otros resultados de interés para el paciente, relacionados con su calidad de vida. A partir de los hallazgos de estas investigaciones, se podría elaborar una propuesta de reforma al sistema de salud que facilite la participación de los pacientes en estudios clínicos con financiación externa (sin transgredir el marco normativo nacional para la investigación en salud con seres humanos), y que impulse la creación de acuerdos beneficiosos entre el Ministerio de Salud y Protección Social y la industria farmacéutica, en los cuales los pacientes siempre sean la prioridad.

La participación de pacientes con cáncer en estudios clínicos evitó costos al sistema de salud de Colombia. El ahorro fue mayor en la atención de mujeres con cáncer de mama entre los 45 y los 59 años y en aquellos pacientes con estadio clínico IV de la enfermedad en el momento del diagnóstico.

Este estudio es el primero en el país que reporta los costos ahorrados al sistema nacional de salud por la participación de los pacientes con cáncer en estudios clínicos financiados por la industria farmacéutica, llevados a cabo en una institución oncológica de cuarto nivel ubicada en una ciudad intermedia de la Región Caribe de Colombia. Además, estos resultados confirman el efecto de apalancamiento de la investigación clínica en los sistemas públicos de salud.
